# Impact of Ultrasound Pre-Treatment on the Drying Kinetics and Quality of Chicken Breast—A Comparative Study of Convective and Freeze-Drying Methods

**DOI:** 10.3390/foods13172850

**Published:** 2024-09-08

**Authors:** Iwona Szymanska, Aleksandra Matys, Katarzyna Rybak, Magdalena Karwacka, Dorota Witrowa-Rajchert, Malgorzata Nowacka

**Affiliations:** 1 Department of Food Technology and Assessment, Institute of Food Science, Warsaw University of Life Sciences–SGGW, 159C Nowoursynowska Street, 02-776 Warsaw, Poland; iwona_szymanska@sggw.edu.pl; 2 Department of Food Engineering and Process Management, Institute of Food Science, Warsaw University of Life Sciences–SGGW, 159C Nowoursynowska Street, 02-776 Warsaw, Poland; aleksandra_matys@sggw.edu.pl (A.M.); katarzyna_rybak@sggw.edu.pl (K.R.); magdalena_karwacka@sggw.edu.pl (M.K.); dorota_witrowa_rajchert@sggw.edu.pl (D.W.-R.)

**Keywords:** ultrasound frequency, ultrasound power, structure, macroscopic changes, rehydration ratio, hygroscopic properties, color

## Abstract

Fresh meat has a limited shelf life and is prone to spoilage. Drying serves as a common method for food preservation. Non-thermal techniques such as ultrasound treatment (US) can positively affect the drying processes and alter the final product. The study aimed to evaluate the impact of US pre-treatment on the hot air (HA) and freeze-drying (FD) of chicken breast meat and the quality of the dried products. US pre-treatment had a varied impact depending on the drying method used. The contact US method extended the HA drying time (about 50%) but improved water removal during FD (about 30%) compared to the untreated samples. Both methods resulted in low water content (<8.3%) and low water activity (<0.44). While rehydration properties (RR) and hygroscopicity (H) were not significantly affected by US pre-treatment in HA drying (about 1.35% and about 1.1, respectively), FD noticed differences due to shrinkage and porosity variations (RR: 2.4–3.2%, H: 1.19–1.25). The HA-dried samples exhibited notably greater tissue shrinkage and a darker surface color than the FD meat. Ultrasonic processing holds substantial potential in creating dried meat products with tailored characteristics. Hence, meticulous consideration of processing methods and parameters is of utmost importance.

## 1. Introduction

Global meat consumption is projected to increase by approximately 14% by 2032 [1]. The worldwide population growth requires an adequate supply of complete protein [2,3]. Animal proteins generally exhibit higher biological value and digestibility than plant proteins [4]. The dominant animal-origin source of protein in the world is poultry meat, estimated to constitute almost half of the additional meat consumed [1]. Poultry meat is valued for many production, nutritional, and consumption reasons [5,6]. Chicken meat is particularly appreciated due to its high production efficiency, favorable composition (low-fat content and high complete protein content), relatively low price, and ease of processing [7,8]. Chicken breast and leg muscles have become the most acceptable to consumers of all communities and, therefore, more economically significant than other parts of the poultry carcass [9]. Thus, chicken meat can be the basic raw material of high-protein products that have become popular recently [10]. Moreover, consumers leading quite intensive lifestyles still expect high-quality convenient food that allows for the easy and quick preparation of meals [11,12]. A significant part of the convenience food market is occupied by dried products, e.g., food concentrates, with a long shelf life [13].

Foods with high water content are perishable and require preservation [14]. Drying is one of the oldest methods of food preservation known to humanity [15]. It facilitates transportation and long-term food storage [16], increases availability, and reduces food waste [17]. Nevertheless, drying is time-consuming and expensive [18]. For this reason, it often constitutes a ‘bottleneck’ in the production. To ensure the microbiological safety of food, it should be carried out as quickly as possible. Therefore, traditional drying methods require some modifications [19,20,21]. Drying at elevated temperatures, e.g., hot air drying, causes relatively significant changes in the quality of the final product, including nutritional losses (e.g., degradation of vitamins, unsaturated fatty acids, and amino acids) and sensorial losses (e.g., deterioration of flavor or color and excessive tissue shrinkage) [8,22]. Low-temperature drying, e.g., freeze-drying, requires increased costs due to lowering and maintaining temperature and pressure [23]. Thus, different pre-treatments can be carried out before drying to enhance the drying rate and/or improve the quality of the dried product. The pre-treatment methods can be divided into chemical and physical groups [24,25]. Emerging non-thermal technologies, such as ultrasounds, high-pressure processing, pulsed electric field, cold plasma, and irradiation, have the potential for sustainable food industry transformation [26,27]. This has contributed to the increased interest in these techniques in food research [28,29,30,31].

One of the effective techniques with high implementation potential is low-frequency high-power ultrasound treatment (US). It causes cavitation (turbulent implosions of bubbles), the “sponge effect” (alternating stresses and relaxations), and produces microchannels, changing the structure of the material, which improves mass and energy transfer [25,30,32]. Therefore, ultrasonic pre-treatment can enhance the drying process by reducing its total operation time (lowering energy consumption) and improving the quality of the dried product. However, studies on the influence of US pre-treatment on the drying process focus mainly on plant raw materials [16,21,33,34,35]. Such findings cannot fully explain the changes occurring in animal tissue because it is a matrix with a different structure and properties than plant tissue. Both preliminary and final processing have a complex impact on the quality of the product. In meat science, there are numerous studies on the effects of ultrasounds on meat alone or in combination with other treatments, e.g., maturation, osmotic dehydration, marination, brining, freezing, and thawing [36,37,38]. There are fewer studies on US pre-treatment and meat drying, and they mainly concern beef [39,40,41,42].

Considering the great potential of the raw material (chicken breast meat), the described technology (US treatment) in producing high-quality dried meat, and the insufficient number of studies, this scientific area was explored. Therefore, this study aimed to investigate the effects of ultrasound pre-treatment on drying kinetics and physicochemical characteristics of chicken breast muscles dried with hot-air and freeze-drying methods. In times of escalating demand for convenience food and an emerging circular economy, ongoing research can hold the potential to advance the field of food science significantly.

## 2. Materials and Methods

### 2.1. Materials

The material used for the research was chicken breast fillet. The meat was produced by DROSED S.A. (Siedlce, Poland) and purchased at a local supermarket. The material was stored under refrigerated conditions (4 ± 1 °C) and used for experiments on the day of purchase. Ten strips were cut from the fillet with a kitchen knife, measuring 8 cm long, 1 cm wide, and 1 cm thick. To ensure homogeneity, samples were consistently extracted from the same breast region.

### 2.2. Ultrasound Pre-Treatment (US)

The pre-treatment involved the application of ultrasound using both immersion (US) and contact (cUS) methods, as detailed in Table 1. The meat samples were weighed using a laboratory scale with an accuracy of 0.01 g. For the immersion method, the meat pieces were submerged in distilled water at a temperature of approximately 4 ± 1 °C, maintaining a water-to-meat mass ratio of 5:1. Ultrasonic cleaners (MKD-3, MKD Ultrasonics, Warsaw, Poland) operating at various frequencies and powers (21 kHz and 300 W; 21 kHz and 180 W; 40 kHz and 180 W) were used. In the case of the contact method, samples were positioned directly on the sonotrode sieve (RIS200, Hielsher Ultrasonics, Teltow, Germany), which was linked to an ultrasound generator (UIS250L, Hielsher Ultrasonics, Teltow, Germany) with a power of 250 W and operating at a frequency of 25 kHz. The treatment was conducted at a consistent temperature of 21 ± 1 °C. The sonication time for both variants was 60 min. After the process, the material was dried on filter paper (in the case of the immersion method) and weighed. Each treatment was replicated three times.

### 2.3. Drying Methods

#### 2.3.1. Hot-Air Drying (HA)

The meat pieces were neatly arranged in a single layer on a perforated metal tray measuring 45 × 30 cm. The drying process was conducted using a prototype dryer (WULS), with an airflow speed of 2 m/s parallel to the material, maintained at 60 °C. Throughout the drying process, the weighing system connected to the tray meticulously recorded the sample mass at 5-min intervals. The drying procedure was performed in three repetitions, and it was concluded when the sample mass exhibited stability for 15 min.

#### 2.3.2. Freeze-Drying (FD)

The meat was rapidly frozen at −40 °C overnight using the HCM 51.20 freezer (Irinox, Treviso, Italy) and then placed on the tray of the Gamma 1–16 LSC laboratory freeze dryer (Martin Christ Gefriertrocknungsanlagen GmbH, Osterode am Harz, Germany). The freeze dryer was equipped with a sample weight recording system (SWL025 Mensor, Warsaw, Poland), which recorded weights every 15 min (every 5 min for the first 120 min). Throughout the process, the shelf temperature was maintained at 40 °C, the pressure was kept at 0.63 mbar, and the condenser temperature was held at −55 °C. The drying process continued until a constant mass was achieved (the same mass was obtained three times). This drying procedure was performed three times.

After the completion of the process, the specimens were taken out of the dryer chambers and placed in PET12/Al8/PE100 (Pakmar, Warsaw, Poland) pouches. These pouches are composed of three layers: polyethylene (12 microns thick) on the outer layer, aluminum (8 microns thick) in the middle, and polyamide (100 microns thick) on the inner layer, providing a solid barrier against water vapor and gases while being utterly impervious to light. The pouches were then sealed and stored at room temperature until needed for analysis. 

Table 2 shows the codes for all (non-treated and pre-treated and raw and dried) samples produced and examined.

### 2.4. Drying Kinetics

Drying kinetics are depicted by the changes in relative water content over the drying process, as described by the following equation:

(1) $$MR\;=\frac{u_{\tau }}{u_{0}},$$

where MR—dimensionless water content [-], u_0_—initial water content [g H_2_O/g d.m.], and u_τ_—water content after time τ [g H_2_O/g d.m.].

The required drying time was determined to achieve MR values of 0.04 and 0.20 based on the drying curves that were obtained.

### 2.5. Measurements of Quality Parameters

#### 2.5.1. Water Content

The water content was determined using the gravimetric drying method by ISO standard (ISO 1442, 2023, [43]). Samples were homogenized for 15 s in an IKA^®^ A11 Basic analytical mill (Darmstadt, Germany). To determine the water content, 5 g of fresh or 0.5 g of dried sample was mixed with 2 g of purified sea sand in a glass vessel. Drying was performed at 103 ± 1 °C for 4 h. Subsequently, the samples were cooled in a desiccator and then weighed. The drying process was repeated for 1 h until a constant weight was achieved. The determination was performed three times.

#### 2.5.2. Water Activity

The water activity (*a_w_*) was determined using an AquaLab Series 3TE instrument (Decagon Devices, Inc., Pullman, Washington, DC, USA), which employs a dew point detection technique on a cooled mirror. Before measurement, the functionality of the device was validated using standards, including 17.18 mol/kg LiCl (0.150 *a_w_*), 6.00 mol/kg NaCl (0.760 *a_w_*), and purified water (1.000 *a_w_*). The measurements were carried out at a temperature of 25 °C with an accuracy of ±0.001. Three repetitions were performed for each type of material.

#### 2.5.3. Macrostructure and Internal Structure

Photographs of the macrostructure of dried meat pieces were captured using a Nikon D-7000 digital camera (Nikon, Tokyo, Japan). The camera was positioned 1 m above the sample, which was placed in a white chamber illuminated by daylight-balanced (5500 K) fluorescent continuous light lamps from four sides. 

Changes in the internal structure of the sample cross-sections were analyzed using a Hitachi TM 3000 tabletop scanning electron microscope (Hitachi, Tokyo, Japan). The microscope had a backscattered electron (BSE) detector [44]. Strips, 2 mm thick, were cut from the dried material and vertically attached with a set screw to a PS12 aluminum pin stub vise clamp. Before analysis, the samples were coated with a 5 nm layer of conductive substance by sputtering them with gold using a Cressington 108 auto sputter coater (Cressington, Watford, UK). The microscope operated in low vacuum mode with an accelerating voltage of 10 kV and magnifications of 100.

#### 2.5.4. Color Parameters

The color was assessed using a benchtop spectrophotometer with a top-facing measurement port (CM-5, Konica Minolta, Osaka, Japan) employing the reflectance method in the CIE Lab* system. The sample was positioned directly above the 8 mm measuring aperture. The observer conditions for the measurement adhered to the CIE standard: 2° observer, standard illuminant (D65), and measurement geometry (d/8°). Each dried product underwent 10 repetitions of measurements. Based on the obtained numerical values for brightness (L*) and two chromatic values (a* and b*), the total color change (Δ*E*) and the browning index (*BI*) were determined using equations [45].

(2) $$∆E=\sqrt{\left(∆L^{*}\right)+\left(∆a^{*}\right)+\left(∆b^{*}\right)}$$

(3) $$BI\;=\;\left(\frac{a^{*}+1.75\;L^{*}}{5.645\;L^{*}+\;a^{*}-3.012\;b^{*}}\;-\;0.31\right)\cdot \frac{100}{0.17}$$

#### 2.5.5. Rehydration Ratio

The rehydration ability of the dry material was assessed by immersing approximately 1 g of dried material in a beaker filled with distilled water at a temperature of 20 °C and rehydration periods for convective dried samples lasted 30, 90, and 180 min. In contrast, lyophilized samples were rehydrated for 5, 15, and 30 min. After the specified duration, the samples were removed, dried, weighed, and their dry substance content was determined. The rehydration coefficient (RR) and the loss of soluble solids (SSL) were calculated using the following equations [46]:

(4) $$RR=\frac{m_{\tau }}{m_{0}}$$

(5) $$SSL=\frac{M_{\tau }{\cdot DM}_{\tau }}{M_{d}{\cdot DM}_{d}}$$

where *m*_τ_—moisture of rehydrated sample at time τ (kg H_2_O/kg d.m.), *m*_0_—initial moisture of dried sample (kg H_2_O/kg d.m.), *M*_τ_—material mass after rehydration time τ (g), *DM*_τ_—dry matter content of sample after rehydration time τ (%), *M_d_*—dried material mass before rehydration (g), and *DM_d_*—dry matter content of dried sample before rehydration (%).

#### 2.5.6. Hygroscopic Properties

The hygroscopic properties of dried chicken meat were evaluated by measuring water vapor sorption. In total, 0.5 g of samples (*m*_0_) were exposed to an environment with a water activity 1.00 (above the pure water) for 24 h at a constant temperature of 25 °C. The weight of the samples (*m*_τ_) was recorded after 1, 3, 6, 9, and 24 h. The hygroscopicity (H) was calculated according to the following formula [47]:

(6) $$H=\frac{m_{\tau }}{m_{0}}$$

### 2.6. Statistical Analyses

The statistical analysis of the results involved a one-way analysis of variance (ANOVA) with the type of treatment as the grouping variable. The hypotheses were confirmed through various tests: normality of data using the Shapiro–Wilk test, equality of variances using the Levene and Brown–Forsythe tests, and equality of means using the sigma-restricted parametrization. In the post-hoc analysis, samples were grouped using the Tukey HSD post-hoc test at a significance level of 0.05 [48]. As a statistical summary of the research, Principal Component Analysis (PCA) was employed to differentiate dried meat samples and establish the relationship between the quality parameters (*n* variables) based on correlation (variances as *SS*/(*n* − 1)). The criteria for selecting the first principal components (factors) were an eigenvalue > 1.0 and a cumulative % of variance > 80%. Factors were correlated with variables, and a significant correlation was indicated when the absolute value of the factor loading was at least 0.7. The correlation coefficients (*r*) of the variables were calculated and interpreted as follows: *r* < ± 0.10 as a negligible correlation; ± 0.10 ≤ *r* ≤ ± 0.39 as a weak correlation; ± 0.40 ≤ *r* ≤ ± 0.69 as a moderate correlation; ± 0.70 ≤ *r* ≤ ± 0.89 as a strong correlation; and *r* ≥ ± 0.90 as a very strong correlation [49]. At the same time, a Hierarchical Cluster Analysis (HCA) was carried out using the agglomeration method (object classification method), the Euclidean distance (distance between objects), the Ward’s method (principle of cluster/object binding), and the Baker and Hubert index (number of cluster) [50]. The mean values and coefficients of variation for each designated cluster [51] were determined using the Statistica 13.1 software (TIBCO Software Inc., Palo Alto, CA, USA).

## 3. Results and Discussion

### 3.1. Influence of Ultrasound Pre-Treatment on the Drying Kinetics of Chicken Breast Meat

**Figure 1:**
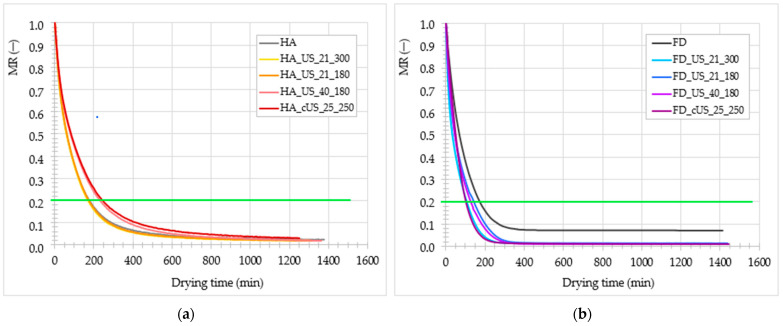
Drying kinetics (dimensionless moisture content—MR) in non-treated and US-treated chicken breast meat dried with (**a**) hot-air (HA) and (**b**) freeze-drying (FD) methods; green line marked on graphs indicates the critical moisture content required for dried meat-based snacks, such as beef jerky, which was established at 0.2.

One of the main purposes of meat pre-treatment using the US is the improvement in mass transfer during processing, which is expected to occur due to structural changes following the treatment. To evaluate the effect of the US on the hot air drying at 60 °C and freeze-drying at 40 °C processes, the kinetics of the moisture content (MR) were determined and presented in Figure 1a,b, respectively. The green line marked on both graphs indicates the critical moisture content required for dried meat-based snacks, such as beef jerky, which was established at 0.2 [52]. On the other hand, using pork and beef as a matrix [53] suggested that the optimum MR for freeze-dried meat was 0.04. Based on that, drying times until the MR in chicken breast meat reached 0.2 and 0.04 were determined for both methods in Table 3. Statistical analysis of the drying times showed that the US only slightly impacted the process, considering that the time required to reach MR equaled 0.2. The application of contact US treatment resulted in a prolongation of the drying time by 39% compared to the control sample (175 min). An increased frequency also caused the HA drying time to rise by 30%. However, it was still in the range similar to the material treated with 21 kHz and the control sample. Comparable dependencies occurred in the following stage of drying that was carried on until the MR was reduced to 0.04 (Figure 1a, Table 3). Compared with the literature regarding plant tissue, the results obtained in this research can be explained by the peculiarities of the material, which is muscle tissue. The fibrous structure of meat absorbs ultrasonic waves, which makes it difficult to exert an effect on the tissue. Moreover, meat lacks air-filled pores, as in plant tissues, resulting in less intense ultrasonic effects [54]. The longer HA drying time might result from structural changes in fibrous structure (see Figure 4), particularly protein, caused by the US. This method of pre-treatment destroys the cellular structure of the muscle. However, it was also observed that the water-holding capacity of US-treated poultry meat increases due to myosin gelling induction [37,55]. Moreover, that phenomenon might have favored crust formation on the surface of the meat, which trapped water inside the material and worsened mass transfer during processing. Additionally, [56] found that the temperature of 70 °C gives better drying rates and quality of the products than 60 or 80 °C. Hence, for further improvement in the HA drying of chicken breast meat, the US pre-treatment may be tested in combination with higher drying temperatures.

In the case of the freeze-drying process, the drying time varied from 181.3 to 267.5 min. The US pre-treatment did not affect the processing time significantly (*p* > 0.05), but the treated material tended to attain the desired moisture content (0.04) faster, especially after subjection to contact treatment. The reduction in the drying time after pre-treatment was 5–32% (Figure 1b, Table 3). 

Faster water removal after the US application was related to the damage made by the electromagnetic waves in the material’s cellular structure. Breaking the internal barriers naturally existing in the tissue made water removal easier. Thus, the drying process was shorter [57]. According to established drying times, freeze-drying provided more effective and faster water removal in the examined material than HA drying. Similar results were obtained before for turkey breast meat [58]. The US treatment was recognized as working appositively in both dehydration methods tested in this study. Freeze-drying combined with the unconventional US pre-treatment gave beneficial and promising results regarding drying kinetics and time, contrary to HA-drying. The most possible explanation is the difference between hot-air and freeze-drying mechanisms. Mass transfer in HA drying is based on the moisture content difference within the sample volume. Water contained in the material migrates to its surface, from which it is removed by the drying agent (hot air). However, exposure to the hot drying agent in HA is associated with structure collapsing and crust formation on the surface of the materials, especially those built out and prone to thermal degradation compounds such as protein. In freeze-drying, moisture removal is driven by a pressure difference between the sample and its surroundings. Moreover, during dehydration, the material is preserved in a frozen state, reducing the risk of unfavorable changes exacerbating the process [57,59].

### 3.2. Influence of Ultrasound Pre-Treatment on the Water Content and Water Activity of Dried Chicken Breast Meat 

Water content and water activity are jointly reliable indicators of the effectiveness of food processing and preservation, as well as its microbiological safety. Raw meat consists of around 75% water. Therefore, the degree and rate of its removal during drying can be criteria for the shelf life of dried meat products [60,61].

The water content and water activity (*a_w_*) in chicken breast samples ranged from 2.81 to 8.20% and 0.099 to 0.436, respectively (Table 4). This is consistent with the requirements for low-moisture foods, which generally contain no more than 25% water [15]. In turn, the water activity for low-moisture food products with extended shelf-life (even without refrigeration) should not exceed 0.6 [62]. Moreover, as seen in Table 4, both the water content and water activity in the FD meat were significantly lower than in the HA-dried samples. During HA drying, meat dehydration is constrained over time by the shrinkage of the muscle myofibril network and connective tissue and, thus, by surface hardening [63,64]. However, during the freeze-drying process, water is gently and gradually removed through micro and macro capillaries of the tissue. The integrity of the muscle fibers is relatively retained, although they become denser and shorter. Importantly, maintaining the porosity of dehydrated muscle tissue increases the effectiveness of its rehydration, which is a relevant quality trait of dried food [23].

The HA-dried meat pre-treated with 300 W ultrasonic power showed about 20% lower water content than the untreated HA-dried sample. Applied ultrasounds could disrupt the cell walls and improve the transfer of water from the intercellular to the extracellular space of the tissue, hence facilitating the further drying process, even with less water activity [37]. In addition, a significant positive linear relationship was found between the water content and water activity of dried chicken meat, which is described by the equation *Wc*% = 11.140 ∙ *a_w_* + 2.5725 and confirmed by the Pearson correlation coefficient *r* = 0.93, *p* = 0001 (Figure S1). However, this relationship is not directly proportional and should be considered individually for each product type [20,22,63]. For example, in this study, HA-dried and FD chicken breast meat pre-treated with 300 W US were statistically similar in water content but significantly different in water activity (Table 4). Importantly, these effects arise from two main factors: the method and parameters of ultrasonic processing, as well as the drying technique. During freeze-drying and hot-air drying, different mechanisms occur and lead to greater or lesser changes in the structure and physical properties of the dried product [34,41,58]. Ultrasound significantly influenced the mass changes in raw chicken breasts even before the drying process. As shown in Table 1, the US contact method resulted in a mass loss, while the US immersion method (regardless of frequency and power) resulted in a mass gain compared to the untreated samples. According to Huang et al. [16] and Ricce et al. [21], the water absorption by tissue during ultrasonic treatment may even hinder its subsequent drying, especially at relatively low temperatures, e.g., ≤ 40 °C. In the present study, this can be observed, for example, in freeze-dried meat pre-treated with ultrasound at a frequency of 21 kHz using the immersion method (Table 1, Table 4). However, it can be stated that the drying method predominantly influenced the water content and water activity of the dried chicken breast meat.

### 3.3. Influence of Ultrasound Pre-Treatment on the Rehydration Ratio and Hygroscopic Properties of Dried Chicken Breast Meat

The process of evaporation of water from the material, commonly known as drying, is associated with the simultaneous occurrence of various biochemical effects, different types of chemical reactions, and modification of the physical properties of the dried material. Physical modifications of the material after drying, e.g., the occurrence and scale of drying shrinkage, decreased or increased porosity, range of water absorption and adsorption capacities (rehydration and hygroscopicity, respectively), and the amount of damage at the microstructural level depend on the characteristics of the matrix, the method of treating the material before drying, and the selected drying method as well as process conditions [65,66,67,68].

**Figure 2:**
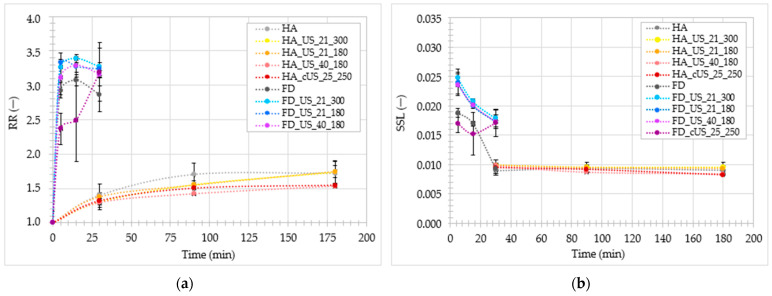
(**a**) Rehydration Ratio (RR), and (**b**) Soluble Solids Loss (SSL) in untreated and US-pretreated hot-air-dried (HA) and freeze-dried (FD) chicken breast meat.

Figure 2a shows how the values of the RR parameter increased depending on the time of immersing dried chicken breast meat in distilled water. As can be seen, at each of the analyzed times, all dried HA samples exhibited a similar ability to absorb water. It increased over time until reaching its maximum after approximately 90 min. Higher variation in RR values occurred in FD samples (Figure 2a). Additionally, regardless of time, soluble solid loss (SSL) in all analyzed dried HA samples remained similar (0.009 ± 0.001—Figure 2b). After 5 and 15 min of analysis, the FD_cUS_25_250 sample showed significantly lower RR and SSL than the other samples, which, as mentioned above, could be due to the more damaged structure of this sample caused by its direct contact with the sonotrode [69,70]. After 30 min of analysis, the untreated FD sample was characterized by a slightly lower RR and SSL than the US-pretreated samples. Taking into account only the analysis time of 30 min, it can be seen that the FD samples had more than twice the values of the RR parameter than the HA samples (Figure 2a,b). As explained above, the reduction in the water absorption capacity of the HA samples could result from the occurrence of shrinkage, and in turn, the significant porosity of the FD samples intensified the water absorption [71,72].

Table 5 contains the rehydration ratio (RR) values obtained by untreated and US-pretreated HA-dried and FD chicken breast meat after 30 min of immersing the materials in distilled water. As can be seen, all samples dried with hot air showed similar rehydration properties, as evidenced by the fact that these samples belong to one homogeneous group. The application of preliminary ultrasonic treatment, regardless of the parameters used or the method of supplying ultrasonic waves to the treated material, did not significantly change the RR of dried HA samples (*p* > 0.05). In the case of FD, all US-pretreated samples did not differ significantly in RR from samples untreated and dried in the same way. However, it was observed that the sample to which ultrasound was delivered in a contact manner (FD_cUS_25_250) exhibited RR lower by 23.8–28.6% than samples treated with the US using water (Table 5). Direct exposure of the material to the sonotrode (contact method) could have led to greater damage to its surface (e.g., to contraction of muscle fibers), which could have contributed to the reduced ability of this sample to reabsorb water [69,70]. Moreover, FD samples had more than twice as high values of the RR parameter as HA samples. This result is the effect of probable differences in the physical properties of the analyzed materials—higher shrinkage of the HA samples, which prevented water absorption, and higher porosity of the FD samples, which enhanced this process [71,72]. 

**Figure 3:**
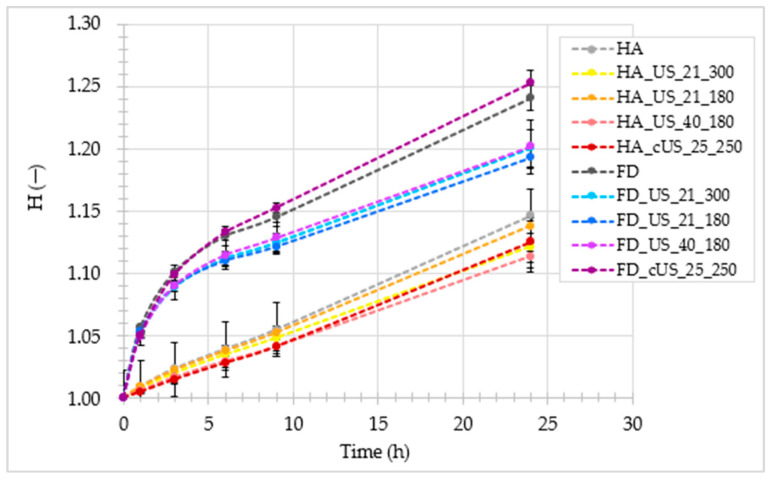
Hygroscopic properties (H) of untreated and US-pretreated hot-air-dried (HA) and freeze-dried (FD) chicken breast meat.

The second important parameter determining the quality of the dried material is hygroscopicity (H). Figure 3 shows the increase in the value of the H parameter of all dried chicken breast meat obtained depending on the time these samples remain above distilled water. At each of the analyzed times, e.g., 0, 1, 3, 6, 9, and 24 h, the HA samples exhibited similar hygroscopicity. Nevertheless, slightly higher H for untreated samples (HA) than US-pretreated samples can be observed. More variation in results occurs in the case of FD drying, especially after 6 h or more of analysis. Samples FD and FD_cUS_25_250, e.g., untreated and treated with contact-supplied US, respectively, achieved noticeably higher values of the H parameter than the other samples. The results may indicate that the disruption of the tissue structure by ultrasound and its transmission through water reduced the treated samples’ ability to adsorb water vapor [73]. 

As shown in Table 5, a one-way analysis of variance showed that neither the application of ultrasound as a preliminary treatment before drying HA and FD (interpreted separately), the modification of the parameters of this process, nor even the change in the method of supplying ultrasonic waves to the treated chicken breast meat, caused statistically significant differences in hygroscopic properties determined after 1 h of testing of the obtained dried materials (*p* > 0.05). In the case of HA drying, this trend also continued after 24 h of analysis. Nevertheless, when analyzing FD drying, a significant difference can be observed in the values of the H parameter (after 24 h of analysis) between the US-treated samples (immersion method) and the US-treated sample (contact method), whose H (1.253 ± 0.011) was comparable to the H achieved by the untreated sample (1.241 ± 0.010). The ability of a given material to adsorb water depends not only on its surface properties but also on its chemical composition. Delivering ultrasonic waves to the material using water could cause significant changes in its chemical characteristics and thus modify its sorption behavior [47]. Similar to the rehydration properties, the FD samples showed higher hygroscopicity than the HA samples, which can also be observed in Figure 3. The obtained results can be explained by the probable differences in the scale of drying shrinkage and porosity of materials dried by both methods [71,72].

### 3.4. Influence of Ultrasound Pre-Treatment on the Structure of Chicken Breast Meat 

**Figure 4:**
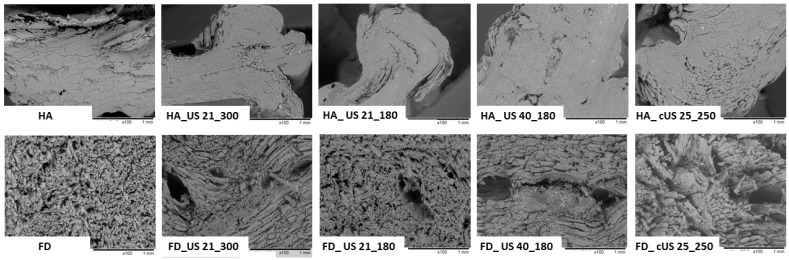
Scanning electron microscope images of untreated and US-pretreated hot-air-dried (HA) and freeze-dried (FD) chicken breast meat, magnification 100×.

**Figure 5:**
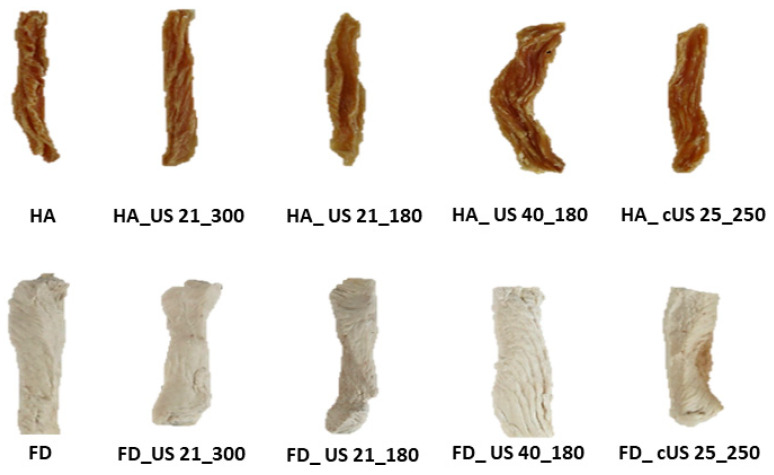
Macroscopic photographs of untreated and US-pretreated hot-air-dried (HA) and freeze-dried (FD) chicken breast meat.

Drying is a complex process in which the material undergoes chemical and physical modifications. Changes in physical characteristics include, among others, shrinkage and porosity [65]. Figure 4 shows images of obtained untreated and US-pretreated HA-dried and FD chicken breast meat taken using the scanning electron microscope. In general, the structure of the samples dried by hot air (regardless of the use of ultrasonic pre-treatment or the differentiation of this process parameters) was relatively dense and compact, with visible shrinkage, which can also be observed on the macroscopic photographs (Figure 5). However, the sample structure to which ultrasound was supplied in a contact manner (HA_cUS_25_250) was slightly more porous than others, characterized by samples that dried similarly. In the case of FD, the effect of ultrasound on chicken breast meat was more visible. One of the effects of ultrasound is the creation of microchannels in the treated tissue [74]. As can be seen in Figure 4, all FD samples with US application before drying had free spaces in their structure. The absence of these channels in the untreated sample (FD) structure was also noticeable. The phenomena that accompany the action of ultrasound, e.g., sponge effect, cavitation, and other accompanying effects, are responsible for the modifications of the treated material. However, their scale depends on the method of supplying ultrasonic waves, selected ultrasonic processing parameters, and the characteristics of the matrix itself [16]. By comparing the images obtained for HA-dried and FD samples, an apparent shrinkage of the former and a much more porous structure can be observed for the latter. This is due to different drying mechanisms. FD involves removing water from the material through the process of ice sublimation. The ice crystals formed in this process compress the material, causing the formation of a porous and spongy structure, a process which, in turn, mitigates the occurrence of drying shrinkage. In turn, during HA drying, water is evaporated from the surface of the dried material, which leads to a pressure difference, and, as a result, the structure collapses [75].

### 3.5. Influence of Ultrasound Pre-Treatment on the Color Parameter of Chicken Breast Meat 

The quality and shelf life of meat is determined by many factors, including animal-specific (e.g., breed, genetics, age, feeding, and pre-slaughter handling), product-specific (e.g., acidity, moisture, texture), process-specific (e.g., ripening, process technology, and heating techniques), and environmental (e.g., temperature, time, packaging, and storage) factors [76]. For consumers, color is still the main indicator of the quality and freshness of meat and the final purchasing decision. Therefore, meat science research frequently examines this quality parameter using objective instrumental analysis methods [77].

Figure 5 shows the visual differences in the color of the HA-dried and FD chicken breast meat samples. Assessing only the appearance of the samples, there was no meaningful effect of US pre-treatment on the color of dried meat, while the drying method caused significant changes. However, the objective evaluation was based on the results of instrumental analysis, expressed in the CIE L*a*b* system, presented in Table 6.

There were no significant differences (*p* > 0.05) in the values of the L* (lightness), a* (redness), and b* (yellowness) color parameters alone between all undried meat samples. The lightness of all the samples, in a range of 53.1–59.1 (Table 6), was typical for fresh chicken breast muscles [7,9]. In turn, the total color difference (Δ*E_RAW_*), ranging from 3.1 to 7.2, showed a quite significant color change in the US pre-treated chicken breasts compared to the untreated one. When ∆*E* exceeds the level of 5, the observer can have an impression of two different colors [78,79]. For example, [36] categorized the total color change in US-treated raw chicken meat as detectable by the human eye at the level of Δ*E* > 5.5. Intriguingly, the slightest color change (Δ*E_RAW_*~3.0) was caused by immersive US treatment at a power of 300 W (Table 6). This is consistent with the findings of [80], who studied the color changes in carp muscles during immersive US-assisted thawing. 

As shown in Table 5, the dried samples exhibited an increase in both a* and b* color coordinates, compared to raw meat, irrespective of US treatment and drying method. In turn, the lightness (L*) of the meat samples notably increased during FD and slightly decreased as a result of HA drying (*p* ≤ 0.05). The variation in color coordinates values has been reflected in the total color difference Δ*E_RAW_*, which varied from 17.0 to 35.4, indicating the perception of two different colors of chicken breast meat. It was concluded that these color changes mainly depended on the drying process and only slightly on the US pre-treatment. It was suggested that the heat generated during ultrasonic processing is insufficient to denature proteins and pigments in meat and, consequently, does not affect its color [81,82]. However, attention was also drawn to the fact that despite a specific total color change in the raw meat caused by ultrasounds, these differences are unnoticeable after thermal treatment [83,84].

When comparing the dried meat, FD samples differed significantly from HA-dried samples in terms of lightness (*p* ≤ 0.05), showing an approximately 2-fold higher value of the L* color parameter. In turn, the HA-dried meat was characterized by a more significant redness (a*) than FD meat (Table 5). As reported by [22], these are the characteristic features differentiating the color of air-dried and freeze-dried meat and meat products. The lighter surface of FD chicken breast meat can be explained by structural changes in myofibrillar and connective tissue proteins caused by their denaturation, leading to optical masking of heme proteins and higher light scattering intensity of the surface. Due to low drying temperature, this phenomenon cannot be explained by the denaturation of heme proteins [5,85] but can be connected with transformations of myoglobin [86]. On the other hand, the increase in redness and darkening of chicken breasts during hot-air drying (Table 5) indicated browning reactions [87] due to high drying temperatures and contact with oxygen-rich air [8], accelerating oxidative reactions, metmyoglobin formation [88], and Maillard reactions [89]. These changes have been visualized numerically using the Browning Index (*BI*), calculated from the L*, a*, and b* color coordinates. As a result of the statistical analysis, HA-dried chicken breasts showed approximately twice the BI value than FD samples. At the same time, ultrasonic pre-treatment did not have any significant effect on the degree of browning of chicken breast samples during drying (*p* > 0.05) (Table 6). However, it was noticed that FD meat pre-treated with contact US treatment at 25 kHz and 250 W was similar to HA-dried meat in *BI* values. In addition, using this method caused the greatest total change in the color of FD meat (Δ*E_FD_*). Presumably, it could be caused by the method of applying ultrasounds that triggered an increase in the sample temperature, which resulted in the adhesion of the meat to the sonotrode sieve. Similar observations were made by [70] when examining the impact of contact ultrasound treatment on apple tissue.

### 3.6. Multivariate Statistical Analysis

**Figure 6:**
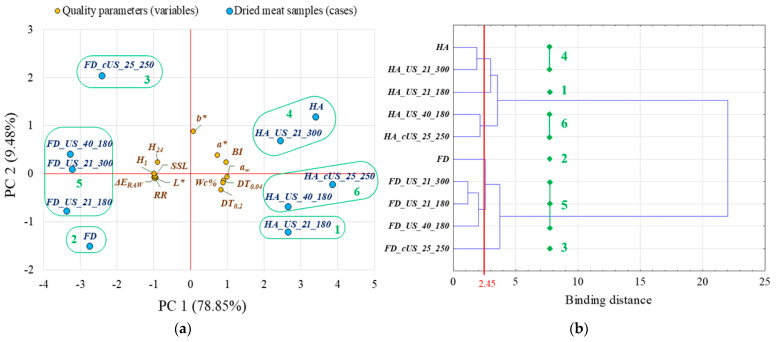
(**a**) Projection of the quality parameters (variables) and the sample types (cases) on the plane of two first principal components and (**b**) a tree diagram derived from Hierarchical Cluster Analysis. For the denomination of the samples, see Table 2. DT0.2—drying time to MR = 0.2 (min), DT0.04—drying time to MR = 0.04 (min), *Wc*%—water content (%), *a_w_*—water activity (─), L*—lightness (─), a*—redness (─), b*—yellowness (─), *BI*—browning index (─), RR—rehydration rate (─), SSL—soluble solid loss (─), H1—hygroscopicity after 1 h (─), H24—hygroscopicity after 24 h (─). The numbers (1–6) presented on graphs shows the separate groups of samples.

Principal Component Analysis led to the extraction of the two first principal components (PCs), factors 1 and 2, which had eigenvalues higher than 1.0 and accounted for 78.85% and 9.48% of the variance, respectively. This means that 88.33% of the total variance for the quality of dried meat in the 13 variables considered can be condensed into two new variables, e.g., PCs. Figure 6a shows that all of the variables had similar proportions in PC1 except for the b* color parameter. HA-dried and FD samples were localized on the positive and negative sides of the PC1 axis, respectively. HA-dried meat pre-treated with contact US contributed most positively along PC1, whereas FD meat pre-treated with immersive US contributed most negatively. The PCA loading plot also suggested the parameters that most contributed to the quality of particular dried chicken breast meat samples. HA-dried meat was positively influenced by drying time, color parameters a* and BI, water activity, and water content. In turn, FD samples were negatively influenced by hygroscopic (*H*_1_, *H*_24_) and rehydration properties (RR, SSL), lightness (L*), and total color difference (Δ*E_RAW_*). The position and angles of the quality parameters (variables) vectors, projected on the plane of the two first principal components, indicate the direction and strengths of the relationships between the variables (Figure 6a). However, the matrix of correlation coefficients and their significance level were examined to illustrate the correlations between variables better, as presented in Table S1. All statistically significant correlations showed at least moderate correlation strength (*r* ≥ 0.4). At the same time, no significant correlation of the b* color coordinate (yellowness) with other quality parameters of dried meat was observed (*p* > 0.05). In turn, the longer the meat is drying, the higher the final water content and water activity of the dried meat (positive correlation), the lower the rehydration capacity, and the worse the hygroscopicity (negative correlation). As previously mentioned, these are characteristics typical of HA-dried meat—Figure 6a. Moreover, the browning index was strongly negatively correlated with the total color difference compared to raw meat (Δ*E_RAW_*), which proves the dominant influence of surface browning on the total change in meat color. 

The results obtained in PCA were reflected in the results of HCA, in which the dried meat variants were divided into six clusters (binding distance: y = 2.45), as shown in the tree diagram in Figure 6b. The clusters were characterized in terms of mean values of the analyzed qualitative parameters (13 variables). HA_US_21_180 (as cluster 1) exhibited the lowest L* and b* color coordinates as well as total color difference compared to raw meat (Δ*E_RAW_*). FD (as cluster 2) showed the lowest browning index, while FD_cUS_25_250 (as cluster 3) had the shortest drying time, the lowest final water content, and the greatest hygroscopicity within 1 h. However, HA and HA_US_21_300 samples (as cluster 4) exhibited the longest drying time until MR = 0.04 and the highest browning index. The other FD meat (FD_US_21_300, FD_US_21_180, and FD_US_40_180) as a cluster 5 were the lightest (L*) and the most different from raw meat in terms of total color change and simultaneously had the highest rehydration rate and soluble solids loss. Cluster 6 involved HA_US_40_180 and HA_cUS_25_250 samples with the longest drying time until MR = 0.2, the highest final water content, and the lowest rehydration rates and hygroscopicity within 1 h. Based on the coefficients of variation (*Vc*), the degree of differentiation of dried meat samples in the cluster concerning a given quality parameter was determined (Table S2). For example, cluster 5 (FD meat pre-treated with immersive US method) and cluster 6 (HA-dried meat pre-treated by US contact method at 25 kHz or US immersion at 40 kHz) were heterogeneous in terms of water content (*Vc* > 20%) and a* color parameter (*Vc* > 30%). Nevertheless, in most cases, *Vc* did not exceed 20%, which indicates a high degree of homogeneity of the clusters identified in this multivariate statistical analysis.

## 4. Conclusions

This study investigated the impact of ultrasound treatment on the drying processes of poultry meat using hot air (HA) and freeze-drying (FD), as well as the quality attributes of the resulting dried meat. The findings revealed that the US treatment had varying effects depending on the drying method. Contact US treatment prolonged the drying time in HA-drying (from about 608 min to about 930 min) due to structural changes in the meat, especially significant shrinkage. Applying the US pre-treatment enhanced FD’s water removal efficiency, reducing drying time by about 30% compared to untreated FD meat. In microscopic observations (SEM), the microchannels formed by the US treatment were more prominent in the FD samples. Additionally, differences in water content, water activity, and color were observed between HA and FD samples, emphasizing the influence of drying methods on product attributes. FD meat exhibited significantly lower final water content (<5.1%) and water activity (<0.14) than HA-dried products. Despite these differences, all the dried meat products met the criteria for low-moisture food, irrespective of the pre-treatment and drying method used. In turn, meat dried using the HA method was characterized by a darker more brownish color compared to FD samples, primarily due to higher temperature processing. In some cases, the ultrasonic treatment slightly affected the color variations in dried meat. Using the contact US pre-treatment method increased the browning of the FD-dried meat compared to the untreated sample. Moreover, rehydration properties and hygroscopicity were minimally affected by US pre-treatment in HA drying, while some changes were noted in FD due to differences in shrinkage and porosity. Applying the hot air drying method led to significant muscle shrinkage, thereby limiting the rehydration of the dried products. Chicken breast meat dried with the freeze-drying method showed a rehydration ratio about twice as high as HA-dried samples. Interestingly, immersion in US pre-treatment reduced the hygroscopicity of FD meat compared to the untreated sample. 

Determining the optimal variant within these studies proves challenging due to the distinct characteristics of the final products. Each tested dried poultry meat sample may exhibit favorable performance in various applications based on specific objectives. The findings underscore the importance of carefully selecting processing methods and parameters to achieve the desired meat products, e.g., jerky snacks with HA-drying application and ingredients to ready-to-eat meals with freeze-drying. 

Furthermore, to better understand the properties of specifically processed poultry meat, it would be beneficial to broaden the study to encompass texture, sensory, and microbiological analyses, especially after rehydration. Furthermore, conducting storage tests of the dried poultry meat under various climatic conditions and utilizing different packaging systems would yield valuable insights into this scientific matter.

## Figures and Tables

**Table 1:** Effect of ultrasound pre-treatment on changes in the mass of chicken breast meat.

Treatment	US Method	Mass Changes (%)	Water Content (%)	Water Activity (─)
Raw	-	-	75.19 ± 0.76 ab	0.954 ± 0.025 a
US_21_300	immersion	3.28 ± 0.35 b	76.41 ± 0.18 bc	0.964 ± 0.016 ab
US_21_180		1.92 ± 0.31 b	77.33 ± 0.16 c	0.979 ± 0.003 b
US_40_180		3.36 ± 1.06 b	76.75 ± 0.78 c	0.979 ± 0.003 b
cUS_25_250	contact	−5.07 ± 1.89 a	74.03 ± 1.01 a	0.973 ± 0.004 ab

Different lowercase letters (a–c) within the same column indicate significant differences between mean values ± standard deviation (*p* ≤ 0.05).

**Table 2:** Codes of the tested samples of chicken breast meat.

Code	US Frequency (kHz)	US Power (W)	Drying Method
Raw	-	-	-
US_21_300	21	300	-
US_21_180	21	180	-
US_40_180	40	180	-
cUS_25_250	25	250	-
HA	-	-	Hot-air drying
HA_US_21_300	21	300	
HA_US_21_180	21	180	
HA_US_40_180	40	180	
HA_cUS_25_250	25	250	
FD	-	-	Freeze-drying
FD_US_21_300	21	300	
FD_US_21_180	21	180	
FD_US_40_180	40	180	
FD_cUS_25_250	25	250	

**Table 3:** The drying time of non-treated and US-treated chicken breast meat dried with hot-air (HA) and freeze-drying (FD) methods to MR = 0.20 and MR = 0.04.

Sample Code	Drying Time (min)	
	MR = 0.20	MR = 0.04
HA	175.0 ± 7.1 bc	607.5 ± 10.6 bc
HA_US_21_300	167.5 ± 24.7 bc	465.0 ± 77.8 b
HA_US_21_180	172.5 ± 3.5 bc	517.5 ± 17.7 bc
HA_US_40_180	227.5 ± 3.5 cd	660.0 ± 56.6 c
HA_cUS_25_250	242.5 ± 10.6 d	930.0 ± 42.4 d
FD	146.9 ± 25.6 ab	267.5 ± 31.8 a
FD_US_21_300	118.8 ± 19.4 ab	222.5 ± 31.8 a
FD_US_21_180	137.5 ± 17.7 ab	253.8 ± 23.0 a
FD_US_40_180	127.5 ± 10.6 ab	245.0 ± 21.2 a
FD_cUS_25_250	102.5 ± 17.7 a	181.3 ± 26.5 a

Different lowercase letters (a–d) within the same column indicate significant differences between mean values ± standard deviation (*p* ≤ 0.05).

**Table 4:** Water content and water activity of untreated and US-pretreated hot-air-dried (HA) and freeze-dried (FD) chicken breast meat.

Sample Code	Water Content (%)	Water Activity (─)
HA	7.39 ± 0.35 ef	0.408 ± 0.039 bc
HA_US_21_300	5.91 ± 1.02 cd	0.353 ± 0.052 b
HA_US_21_180	6.91 ± 0.13 def	0.386 ± 0.028 bc
HA_US_40_180	6.17 ± 0.44 cde	0.399 ± 0.039 bc
HA_cUS_25_250	8.20 ± 0.39 f	0.436 ± 0.035 c
FD	3.87 ± 0.47 ab	0.101 ± 0.024 a
FD_US_21_300	5.03 ± 0.68 bc	0.130 ± 0.030 a
FD_US_21_180	4.46 ± 1.43 b	0.106 ± 0.041 a
FD_US_40_180	2.82 ± 0.49 a	0.084 ± 0.028 a
FD_cUS_25_250	2.81 ± 0.40 a	0.099 ± 0.028 a

Different lowercase letters (a–f) within the same column indicate significant differences between mean values ± standard deviation (*p* ≤ 0.05).

**Table 5:** Rehydration ratio (RR) and hygroscopic properties (H) of untreated and US-pretreated hot-air-dried (HA) and freeze-dried (FD) chicken breast meat.

Sample Code	RR (%)	H (─)	
	After 30 min	After 1 h	After 24 h
HA	1.40 ± 0.13 a	1.009 ± 0.002 a	1.146 ± 0.011 a
HA_US_21_300	1.30 ± 0.11 a	1.007 ± 0.002 a	1.121 ± 0.021 a
HA_US_21_180	1.38 ± 0.05 a	1.008 ± 0.001 a	1.138 ± 0.006 a
HA_US_40_180	1.30 ± 0.04 a	1.005 ± 0.002 a	1.114 ± 0.005 a
HA_cUS_25_250	1.32 ± 0.04 a	1.004 ± 0.001 a	1.125 ± 0.020 a
FD	2.92 ± 0.11 bc	1.056 ± 0.003 b	1.241 ± 0.010 c
FD_US_21_300	3.26 ± 0.20 c	1.051 ± 0.002 b	1.201 ± 0.015 b
FD_US_21_180	3.32 ± 0.25 c	1.053 ± 0.002 b	1.193 ± 0.009 b
FD_US_40_180	3.11 ± 0.24 c	1.049 ± 0.007 b	1.202 ± 0.022 b
FD_cUS_25_250	2.37 ± 0.22 b	1.050 ± 0.003 b	1.253 ± 0.011 c

Different lowercase letters (a–c) within the same column indicate significant differences between mean values ± standard deviation (*p* ≤ 0.05).

**Table 6:** Color parameters of chicken breast meat undried and dried with hot-air (HA) and freeze-drying (FD) methods.

Sample Code	L* (─)	a* (─)	b* (─)	*BI* (─)	Δ*E_RAW_* (─)	Δ*E_HA/FD_* (─)
RAW	53.1 ± 1.9 ab	−1.9 ± 0.4 a	4.0 ± 1.7 a	-	-	-
US_21_300	54.9 ± 4.4 ab	−2.7 ± 0.3 a	1.4 ± 0.2 a	-	3.1 ± 0.2 a	-
US_21_180	59.1 ± 5.4 bc	−2.5 ± 0.5 a	2.4 ± 0.9 a	-	6.9 ± 3.8 ab	-
US_40_180	59.0 ± 4.9 bc	−2.5 ± 0.6 a	1.0 ± 2.1 a	-	7.2 ± 1.5 ab	-
cUS_25_250	53.4 ± 4.9 ab	−2.3 ± 0.5 a	3.7 ± 3.8 a	-	4.9 ± 2.0 a	-
HA	44.4 ± 5.2 ab	7.2 ± 1.5 c	18.5 ± 1.3 b	65.0 ± 5.3 d	20.1 ± 1.3 cd	-
HA_US_21_300	44.4 ± 6.7 ab	4.8 ± 1.6 bc	18.1 ± 1.3 b	59.2 ± 7.5 d	18.5 ± 1.7 cd	3.9 ± 0.2 a
HA_US_21_180	43.3 ± 5.9 a	5.3 ± 1.8 bc	15.1 ± 1.1 b	51.2 ± 2.1 cd	17.0 ± 0.3 cd	6.2 ± 1.0 ab
HA_US_40_180	48.2 ± 6.3 ab	3.1 ± 1.6 b	17.4 ± 1.2 b	48.5 ± 0.7 bcd	16.3 ± 0.2 bc	5.1 ± 0.3 a
HA_cUS_25_250	43.3 ± 5.3 a	4.8 ± 2.2 bc	18.0 ± 0.5 b	60.8 ± 3.5 d	19.0 ± 1.0 cd	4.8 ± 2.0 a
FD	81.0 ± 7.1 d	2.9 ± 1.7 b	15.2 ± 4.2 b	23.7 ± 10.3 a	31.2 ± 5.6 e	-
FD_US_21_300	82.1 ± 3.1 d	1.9 ± 0.9 b	18.2 ± 2.1 b	26.2 ± 3.6 a	33.8 ± 0.0 e	7.6 ± 2.3 ab
FD_US_21_180	84.5 ± 3.7 d	2.0 ± 1.2 b	16.9 ± 0.3 b	23.5 ± 0.2 a	35.4 ± 0.4 e	5.2 ± 0.1 a
FD_US_40_180	79.3 ± 7.9 d	3.5 ± 2.6 b	17.4 ± 2.0 b	28.0 ± 8.1 ab	31.5 ± 4.4 e	9.6 ± 6.8 ab
FD_cUS_25_250	71.6 ± 6.7 cd	4.1 ± 2.0 bc	19.1 ± 1.2 b	34.6 ± 0.8 abc	26.1 ± 2.3 de	16.9 ± 2.0 b

BI—Browning Index; Δ*E_RAW_*—total color difference compared to raw chicken breast meat; Δ*E_HA/FD_*—total color difference compared to non-treated chicken breast meat dried with hot-air or freeze-drying method; Different lowercase letters (a–d) within the same column indicate significant differences between mean values ± standard deviation (*p* ≤ 0.05).

## Data Availability

The original contributions presented in the study are included in the article/supplementary material; further inquiries can be directed to the corresponding author.

## Supplementary Materials

The following supporting information can be downloaded at https://www.mdpi.com/article/10.3390/foods13172850/s1: Figure S1: Correlation curve between water content and water activity of hot-air (HA) dried and freeze-dried (FD) chicken breast meat; Table S1: Matrix of correlation coefficients (r) for the variables of hot-air (HA) dried and freeze-dried (FD) chicken breast meat; and Table S2: Characteristics of groups (clusters) of dried meat samples as a result of Hierarchical Cluster Analysis (HCA).
