# Supplementary material for: Impact of Ultrasound Pre-Treatment on the Drying Kinetics and Quality of Chicken Breast—A Comparative Study of Convective and Freeze-Drying Methods
Source: Foods. 2024 Sep 8;13(17):2850. doi: 10.3390/foods13172850 (PMC11395696; doi:10.3390/foods13172850)
Supplement: Supplementary file 1 [file foods-13-02850-s001.zip › foods-3191153-supplementary.pdf]

Supplementary material

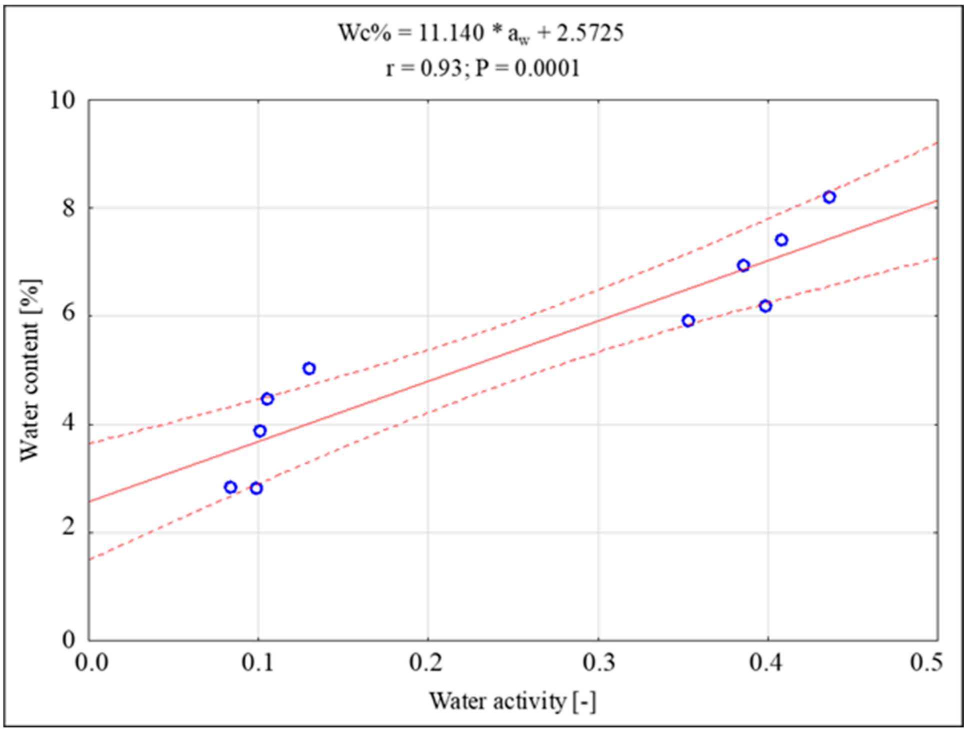

Figure S1. Correlation curve between water content and water activity of hot-air dried (HAD) and freeze-dried (FD) chicken breast meat

Table S1. Matrix of correlation coefficients (*r*) for the variables of hot-air dried (HAD) and freeze-dried (FD) chicken breast meat.

Variable	$DT_{0.2}$	$DT_{0.04}$	Wc%	a_w	L*	a*	b*	BI	ΔE_{RAW}	RR	SSL	H_1	H_{24}
$DT_{0.2}$	1.00												
$DT_{0.04}$	0.96	1.00											
Wc%	0.82	0.89	1.00										
a_w	0.86	0.91	0.93	1.00									
L*	-0.76	-0.84	-0.83	-0.97	1.00								
a*	0.34	0.53	0.56	0.67	-0.77	1.00							
b*	-0.08	0.07	0.00	0.06	-0.10	0.18	1.00						
BI	0.70	0.83	0.83	0.94	-0.97	0.84	0.27	1.00					
ΔE_{RAW}	-0.73	-0.78	-0.72	-0.92	0.97	-0.70	-0.08	-0.90	1.00				
RR	-0.75	-0.82	-0.77	-0.94	0.99	-0.74	-0.11	-0.94	0.99	1.00			
SSL	-0.75	-0.81	-0.76	-0.92	0.97	-0.77	-0.04	-0.93	0.96	0.99	1.00		
H_1	-0.82	-0.88	-0.87	-0.99	0.98	-0.68	-0.09	-0.95	0.95	0.96	0.93	1.00	
H_{24}	-0.74	-0.75	-0.89	-0.91	0.85	-0.59	0.15	-0.83	0.77	0.81	0.82	0.89	1.00

For denomination of the variables, see Figure 7. Correlation coefficients $r > 0$, $r < 0$ or $r = 0$ indicate positive, negative or no relationship between two variables, respectively ($P \leq 0.05$). Correlation strength: nonsignificant ($P > 0.05$); $r < \pm 0.10$ as a negligible correlation; $\pm 0.10 \leq r \leq \pm 0.39$ as a weak correlation; $\pm 0.40 \leq r \leq \pm 0.69$ as a moderate correlation; $\pm 0.70 \leq r \leq \pm 0.89$ as a strong correlation; $r \geq \pm 0.90$ as a very strong correlation .

Table S2. Characteristics of groups (clusters) of dried meat samples as a result of Hierarchical Cluster Analysis (HCA)

Cluster	Sample	Value	$DT_{0.2}$ [min]	$DT_{0.04}$ [min]	Wc% [%]	a_w [-]	L* [-]	a* [-]	b* [-]	BI [-]	ΔE_{RAW} [-]	RR [-]	SSL [-]	H_1 [-]	H_{24} [-]
1	HA_US_21_180	$\bar{x}$	172.5	517.5	6.9	0.4	43.3	5.3	15.1	51.2	17.0	1.4	0.010	1.01	1.12
		sd	-	-	-	-	-	-	-	-	-	-	-	-	-
		Vc	-	-	-	-	-	-	-	-	-	-	-	-	-
2	FD	$\bar{x}$	146.9	267.5	3.9	0.1	81.0	2.9	15.2	23.7	32.1	2.9	0.019	1.06	1.19
		sd	-	-	-	-	-	-	-	-	-	-	-	-	-
		Vc	-	-	-	-	-	-	-	-	-	-	-	-	-
3	FD_cUS_25_250	$\bar{x}$	102.5	181.3	2.8	0.1	71.6	4.1	19.1	34.6	26.1	2.4	0.017	1.05	1.25
		sd	-	-	-	-	-	-	-	-	-	-	-	-	-
		Vc	-	-	-	-	-	-	-	-	-	-	-	-	-
4	HA<br>HA_US_21_300	$\bar{x}$	171.3	536.3	6.7	0.4	44.4	6.0	18.3	62.1	19.3	1.4	0.009	1.01	1.12
		sd	5.3	100.8	1.0	0.0	0.0	1.7	0.3	4.1	1.2	0.1	0.0	0.0	0.0
		Vc	3%	19%	16%	10%	0%	28%	2%	7%	6%	5%	7%	0%	0%
5	FD_US_21_300<br>FD_US_21_180<br>FD_US_40_180	$\bar{x}$	127.9	240.4	4.1	0.1	82.0	2.5	17.5	25.9	33.6	3.2	0.024	1.05	1.21
		sd	9.4	16.1	1.1	0.0	2.6	0.9	0.6	2.3	2.0	0.1	0.0	0.0	0.0
		Vc	7%	7%	28%	22%	3%	36%	4%	9%	6%	3%	2%	0%	2%
6	HA_US_40_180<br>HA_cUS_25_250	$\bar{x}$	235.0	795.0	7.2	0.4	45.7	3.9	17.7	54.7	17.7	1.3	0.010	1.00	1.14
		sd	10.6	190.9	1.4	0.0	3.5	1.2	0.5	8.7	1.9	0.0	0.0	0.0	0.0
		Vc	5%	24%	20%	6%	8%	31%	3%	16%	11%	1%	3%	0%	1%

For denomination of the: samples - see Table 2; variables - see Figure 7. $\bar{x}$ - mean value, sd – standard deviation from the $\bar{x}$ value, Vc – coefficient of variation
